# Peptide Profiling of Young Gouda Holland Cheese and Its Digests Obtained by In-Vitro-Simulated Gastrointestinal Digestion

**DOI:** 10.1021/acs.jafc.5c04616

**Published:** 2025-08-11

**Authors:** Débora Parra Baptista, Sevim Dalabasmaz, Sabrina Gensberger-Reigl, Mirna Lúcia Gigante, Monika Pischetsrieder

**Affiliations:** † Food Chemistry, Department of Chemistry and Pharmacy, Friedrich-Alexander-Universität Erlangen-Nürnberg (FAU), Nikolaus-Fiebiger-Straße 10, 91058 Erlangen, Germany; ‡ Department of Food Engineering and Technology, School of Food Engineering, Universidade Estadual de Campinas, UNICAMP, 13083-862 Campinas, SP, Brazil

**Keywords:** proteolysis, casein, gastrointestinal digestion, bioactive peptides

## Abstract

Ripened cheeses contain bioactive peptides that are released from caseins by enzymatic hydrolysis during fermentation and ripening. However, the physiological relevance of these peptides depends on their stability during gastrointestinal digestion and their bioavailability. This study aimed to assess the impact of digestion on the peptide profile of the young Gouda Holland cheese. Using liquid chromatography coupled to electrospray ionization–tandem mass spectrometry (LC-ESI-MS/MS), we monitored the peptide profile changes during in vitro simulated gastrointestinal digestion. In total, 54 peptide sequences were identified in cheese (20), gastric (20), and intestinal digests (24). Ten peptides were derived from α_s1_-casein, 43 from β-casein, and 1 from κ-casein. Most of the identified peptides were exclusive to one of the analyzed samples, revealing the alteration of the peptide profile during digestion. Two peptides were resistant to digestion, including β-CN­(f193–209) with reported antithrombin, antimicrobial, and ACE-inhibitory effects. These results demonstrate the dual role of digestion in both degrading and releasing bioactive peptides and emphasize the importance of using digestion models to assess the bioactive potential of peptides in dairy products.

## Graphical abstract

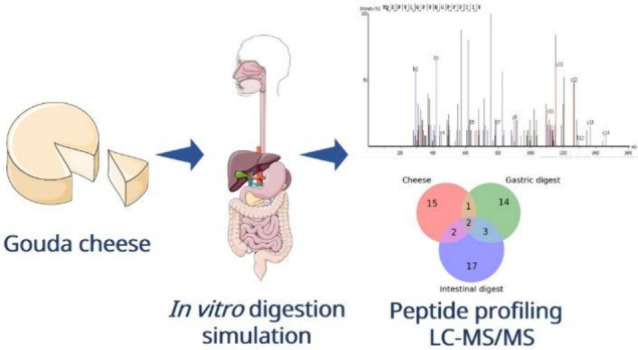

## 1 Introduction

Gouda is a traditional Dutch ripened cheese produced from a washed curd obtained by enzymatic coagulation of pasteurized bovine milk. It is characterized by a semihard to semisoft consistency with a few small round holes and a wide variation in flavor intensity and profile depending on cheesemaking and ripening conditions.^1^

In 2010, the European Commission approved the registration of protected designations of origin and protected geographical indications to “Gouda Holland”, the traditional Gouda cheese produced exclusively in the Netherlands from pasteurized bovine milk from Dutch dairy farms. Gouda Holland cheese is manufactured under a series of specified conditions and must be naturally ripened for at least 28 days at a minimum temperature of 12 °C.^2^

The characteristic flavor and texture of a cheese variety is achieved by several biochemical reactions that occur during ripening: glycolysis, catabolism of lactate and citrate, lipolysis, catabolism of free fatty acids, proteolysis, and catabolism of amino acids.^3^ Proteolysis is considered to be the most complex of all biochemical changes that occur during cheese ripening. It is characterized by several reactions that lead to the release of peptides with different chain lengths and also amino acids.^4^

Proteolysis plays an essential role in the development of ripened cheese characteristics, leading to changes in the cheese texture and flavor attributes. This process is driven by proteases and peptidases from different origins, such as native milk enzymes, residual coagulant, enzymes from starter and nonstarter bacteria, adjunct cultures, and exogenous enzymes.^4^ In addition to contributing to sensory aspects, many peptides released during cheese ripening are also recognized as bioactive peptides and may have several physiological effects, including antimicrobial, antioxidant, opioid, and antihypertensive activities.^5–11^

Bioactive peptides in Gouda have been previously reported.^9,12,13^ However, the presence of bioactive peptides in cheese does not guarantee that these peptides always exert their beneficial effects when the product is ingested. During cheese digestion, protein and peptide breakdown occur due to the action of digestive enzymes. Thus, new biopeptides may be released and active peptides might be hydrolyzed, leading to the release of fragments without physiological activity.^10,11,14–17^

In this context, the aim of this study was to monitor the peptide profile of the Gouda Holland cheese during the different phases of gastrointestinal digestion. For this purpose, Gouda gastrointestinal digestion was simulated using the in vitro static harmonized protocol proposed by the COST INFOGEST international cooperation network.^18^ The peptide profiles of cheese and digests obtained during simulated digestion were recorded by ultrahigh-performance liquid chromatography hyphenated electrospray ionization tandem mass spectrometry to reveal the changes in the peptide profiles.

## 2 Material and Methods

### 2.1 Cheese Samples

Three different batches of commercial Gouda Holland young (4 weeks of ripening) from the same brand were purchased from a local German supermarket. Each cheese sample consisted of a 375 g vacuum-packed slice. According to the product label, the cheese contained 31% fat, 24% protein, 1.6% salt, and less than 0.1% carbohydrate. Prior to analysis, the cheese rind and the surface of each slice were removed. Then, the cheeses were ground using a grinding instrument (Grindomix GM200, Retsch GmbH, Haan, Germany) and frozen (−20 °C) until digestion and peptide profiling.

### 2.2 Simulated Gastrointestinal Digestion of Cheese Samples

In vitro digestion was performed with the commercial Gouda Holland young cheese according to the harmonized protocol proposed by the international scientific cooperation network INFOGEST that simulates the gastrointestinal digestion based on available physiological data.^18^ Briefly, 5 g of ground cheese sample or 5 mL of ultrapure water as a blank control were mixed with 4 mL of simulated salivary stock solution (15.1 mM KCl, 3.7 mM KH_2_PO_4_, 13.6 mM NaHCO_3_, 0.15 mM MgCl_2_, 0.06 mM (NH_4_)_2_CO_3_), 25 μL of 0.3 M CaCl_2_ and 975 μL of ultrapure water. The mixture of cheese and fluids was homogenized using an Ultraturrax (IKA, T18, Staufen, Germany) to obtain a thin paste-like consistency. Amylase was not included in the oral phase of the digestion since cheese does not contain starch. The mixture was incubated for 2 min at 37 °C and 120 rpm in an incubator shaker (New Brunswick, Nürtingen, Germany) to simulate the oral phase. Then, 7.5 mL of simulated gastric stock solution (6.9 mM KCl, 0.9 mM KH_2_PO_4_, 25 mM NaHCO_3_, 47.2 mM NaCl, 0.1 mM MgCl_2_, 0.5 mM (NH_4_)_2_CO_3_), 1.6 mL of pepsin solution (Sigma-Aldrich, P7000, 25,000 U mL^–1^), and 5 μL of 0.3 M CaCl_2_ were added. The pH was adjusted to 3.0 with 1 M HCl, and ultrapure water was added to achieve a final volume of 10 mL of fluid in the gastric phase. The mixture was incubated at 37 °C for 2 h at 120 rpm to simulate the gastric phase. Finally, 11 mL of simulated intestinal stock solution (6.8 mM KCl, 0.8 mM KH_2_PO_4_, 85 mM NaHCO_3_, 38.4 mM NaCl, 0.33 mM MgCl_2_), 5 mL of pancreatin solution (Sigma-Aldrich, P1750, 800 U mL^–1^), 2.5 mL of bile solution (Sigma-Aldrich, B8631, 160 mM) and 40 μL of 0.3 M CaCl_2_ were added. The pH was adjusted to 7.0 with 1 M NaOH, and ultrapure water was added to achieve a final volume of 20 mL of fluids added in the intestinal phase. The mixture was incubated at 37 °C for 2 h at 120 rpm to simulate the intestinal phase. To obtain gastric digest and intestinal digest samples, the experiment was conducted in parallel with 2 incubation flasks. After the gastric phase, the pH was adjusted to 7.0 with 1 M NaOH in one flask to inactivate the pepsin. This sample was immediately frozen in liquid nitrogen. The second flask was used for intestinal digestion. After intestinal digestion, the pH was adjusted to 11.0 with 1 M NaOH to inactivate the pancreatin and was immediately frozen in liquid nitrogen. The samples were stored at −20 °C until peptide profiling. The complete procedure was repeated in three independent triplicates.

### 2.3 Peptide Extraction from Cheeses and Digests

The Gouda digests were defrosted, and all the volumes were adjusted to a volume of 40 mL to standardize the ratio of substrate (cheese) and digestion fluids. For comparison of the peptide profile of the cheese sample and digests, 5 g of cheese was homogenized with ultrapure water using an Ultraturrax (IKA, T18, Staufen, Germany) and diluted to a final volume of 40 mL. Samples were defatted twice by centrifugation at 1400*g* for 30 min and 4 °C and the soluble phase was ultrafiltered using ultrafiltration centrifugal units with 10 kDa cutoff and modified poly­(ether sulfone) membrane material (Pall Corporation, New York, USA) at 4 °C and 2370*g*, and the pH of all digests was adjusted to 7.0.

Finally, peptides were extracted from the aqueous cheese extracts and digestion filtrates by StageTip microextraction according to Rappsilber et al.^19^ modified by Baum et al.^20^ and Ebner et al.,^21^ with further modifications. Briefly, to prepare the StageTips, 1 mm diameter discs were punched from an Empore C18 extraction disc (3M, Catalog No. 2215, Neuss, Germany) using a biopsy punch (Kai Industries Co., Japan). Three discs were stacked sequentially into a 200 μL pipet tip, which was then fitted into the perforated cap of a 2 mL microcentrifuge tube. The StageTip was first equilibrated with 100 μL of acetonitrile (ACN), followed by centrifugation at 2370*g* for 1 min, and 100 μL of 0.1% formic acid (FA), followed by centrifugation at 2370*g* for 1 min. Then, 40 μL of the ultrafiltrate of the water-soluble extract was loaded onto the StageTip, followed by centrifugation at 4650*g* for 5 min. The tip was then washed with 50 μL of 0.1% FA by centrifugation at 2370*g* for 3 min. Finally, peptides were eluted with 10 μL of 60% ACN in 0.1% FA followed by centrifugation at 2370*g* for 3 min. The eluates were stored at −20 °C for further use. As the sample volumes were equalized after digestion and the peptide analysis was qualitative rather than quantitative, peptide concentrations were not measured after extraction.

### 2.4 Peptide Profiling by UHPLC-ESI-QTRAP-MS/MS

The peptide profiling of the samples and their digests was performed on a Dionex Ultimate 3000 RS system (ThermoFisher, Dreieich, Germany), coupled to a Sciex 4000 QTrap mass spectrometer (Sciex, Darmstadt, Germany), equipped with an ESI source (Turbo V, Darmstadt, Germany). The peptide mixtures obtained by StageTip extraction were diluted 1:30 with 0.1% FA in ultrapure water (eluent A). A portion of 10 μL was injected onto a C18 column (Waters Acquity UPLC Peptide CHS C18; 2.1 × 100 mm, 1.7 μm) with a flow rate of 0.3 mL/min and a column temperature of 30 °C. The column was equilibrated for 6 min with a mixture of 95% eluent A (0.1% FA) and 5% eluent B (ACN). The chromatographic separation of the peptides was carried out using the following gradient: 0 min, 5% B; 5 min, 5% B; 55 min, 50% B; 56 min, 90% B; and 60 min, 90% B. All flow eluting between 2 and 55 min was directed into the mass spectrometer by a two-position valve. The MS measurements were performed in the positive mode. The ion source was operated at 500 °C with a voltage of 5000 V and a declustering potential of 50 V. The curtain gas was set to 50 psig, the nebulizer gas to 60 psig, and the heating gas to 75 psig. A scan range of 150–1500 *m*/*z* was used with a scan rate of 1000 Da/s. All mass spectra were acquired using QTRAP-enhanced full mass scan mode (EMS). As the 4000 QTRAP system is a hybrid triple quadrupole LIT (linear ion trap) mass spectrometer, its third quadrupole can be operated as a linear ion trap mass spectrometer to provide EMS scans with high sensitivity. EMS measurements were carried out in triplicate from independent StageTip extractions.

### 2.5 Peptide Identification by MicroLC-ESI-QTRAP-MS/MS

The samples were additionally analyzed with a UHLPC system coupled to a QTRAP 6500^+^ mass spectrometer equipped with an IonDrive Turbo V source. An aliquot of the StageTip extract was diluted 1:30 with 0.1% FA in ultrapure water (eluent A). A portion of 5 μL was injected into a C18 column (YMC Triart, 500 μm × 100 mm, 3 μm; YMC Europe GmbH, Dinslaken, Germany) at a flow rate of 30 μL/min and a column oven temperature of 35 °C. Chromatographic separation of the peptides was achieved by applying a gradient of 0.1% FA as eluent A and 0.1% FA in ACN as eluent B (−15 min 2% B, 5 min 2% B, 55 min 42.5% B, 55.5 min 95% B, and 65 min 95% B). The LC flow between 2 and 55 min was led into the mass spectrometer. The ion source was operated with a voltage of 5500 V and a temperature of 350 °C. The curtain gas was set to 20 psig and the nebulizer gas to 11 psig. The declustering and entrance potentials were set as 80 and 10 V. Information-dependent acquisition (IDA) was performed in positive mode in the mass range of 150–1500 Da. Up to eight most intense ions that exceed 1000 and 10,000 cps with the charge state 1 to 3 were included. Rolling collision energy was used for collision-induced fragmentation. Former target ions were excluded after one occurrence for 10 s with a mass tolerance of 250 mDa and the isotopes within four Da. An enhanced resolution (ER) scan was used to confirm the charge state of the ions. Scan rates for EMS, ER, and enhanced product ion (EPI) scan modes were 1000, 250, and 10,000 Da/s, respectively.

MS/MS spectra from the IDA experiments were searched using PEAKS X software (Bioinformatics Solutions Inc., Waterloo, Ontario, Canada) against the Uniprot-Swiss-Prot database, with selected as the taxonomy. The error tolerance of precursor mass using monoisotopic mass and for the fragment ions was 0.5 Da. Enzymes were specified by each sample, and a semispecific digest mode was used. A multiple-enzyme approach was used, considering pepsin for gastric digests and both trypsin and chymotrypsin for intestinal digests. Three maximum missed cleavages and three variable post-translational modifications were allowed per peptide. Acetylation, amidation, deamidation, oxidation, phosphorylation, and pyroglutamate (pyroQ) formation from Q or E were set as variable modifications. False discovery rate (FDR) calculation was enabled. The PEAKS peptide score (−10lgP) was set as −10lgP ≥ 20. The score is derived from the *p*-value, indicating the statistical success of the peptide-spectrum match.^22^ When the −10lgP value is equal to or higher than 20, the *p*-value corresponds to 0.01 or lower values, respectively. The proposed peptide sequences were manually verified by inspecting the quality of the matched signals.

### 2.6 Database Search for Bioactive Peptides and Enzymes Cleavage Sites

An in silico search for potential bioactivities was performed for all peptides identified, in cheese and digests, using the amino acid sequences in single-letter codes as search terms. The search was conducted against the Milk Bioactive Peptide Database (MBPDB; http://mbpdb.nws.oregonstate.edu),^23^ as well as through literature searches in Google Scholar, PubMed, and Web of Knowledge. For all searches, only exact matches between the sequences were considered, and the original references for each determined bioactivity were verified.

To better understand the release of peptides in cheese, the cleavage sites of the main enzymes acting in cheese ripening in each protein sequence were searched in the literature.^4,24^ To better understand the origin of the peptides in the digests, the bioinformatic tool Peptide Cutter^25^ was used to predict the cleavage sites of pepsin, trypsin, and chymotrypsin in each casein fraction's amino acid sequence.

## 3 Results and Discussion

### 3.1 Peptide Profiling of Cheeses and Cheese Digests

The present study focused on the peptide profile of commercial Gouda Holland cheese and changes in the profile during simulated gastrointestinal digestion. For this purpose, first, peptide profiles of three different batches of Gouda young cheeses were recorded by UHPLC–ESI–MS. Due to the similarity between the peptide profiles of the different batches (see Supporting Information, Figure S1), only one batch was used for the simulated gastrointestinal digestion, and the complete experiment was repeated in triplicate. Gastrointestinal digestion of the cheese resulted in changes in the peptide profile due to the action of the digestive enzymes pepsin and pancreatin. The comparison of the total ion chromatograms of the cheese, gastric digests, and intestinal digests showed clear differences in the peptide profile (Figure 1). While in the cheese sample, the most intense signals appeared between 13 and 16 min (13.8, 14.5, and 15.8 min), in the gastric digest, these signals were less abundant, and a higher response was observed from 15 to 30 min, with one major signal at 30.4 min. Several intense peaks eluting between retention times 35 and 55 min were detected in the intestinal digest. However, most of these abundant peaks were also detected in the blank intestinal sample and are due to the presence of bile salts in the intestinal phase of digestion. Peaks corresponding to bile salt signals were marked with an arrow in Figure 1C.

**Figure 1:**
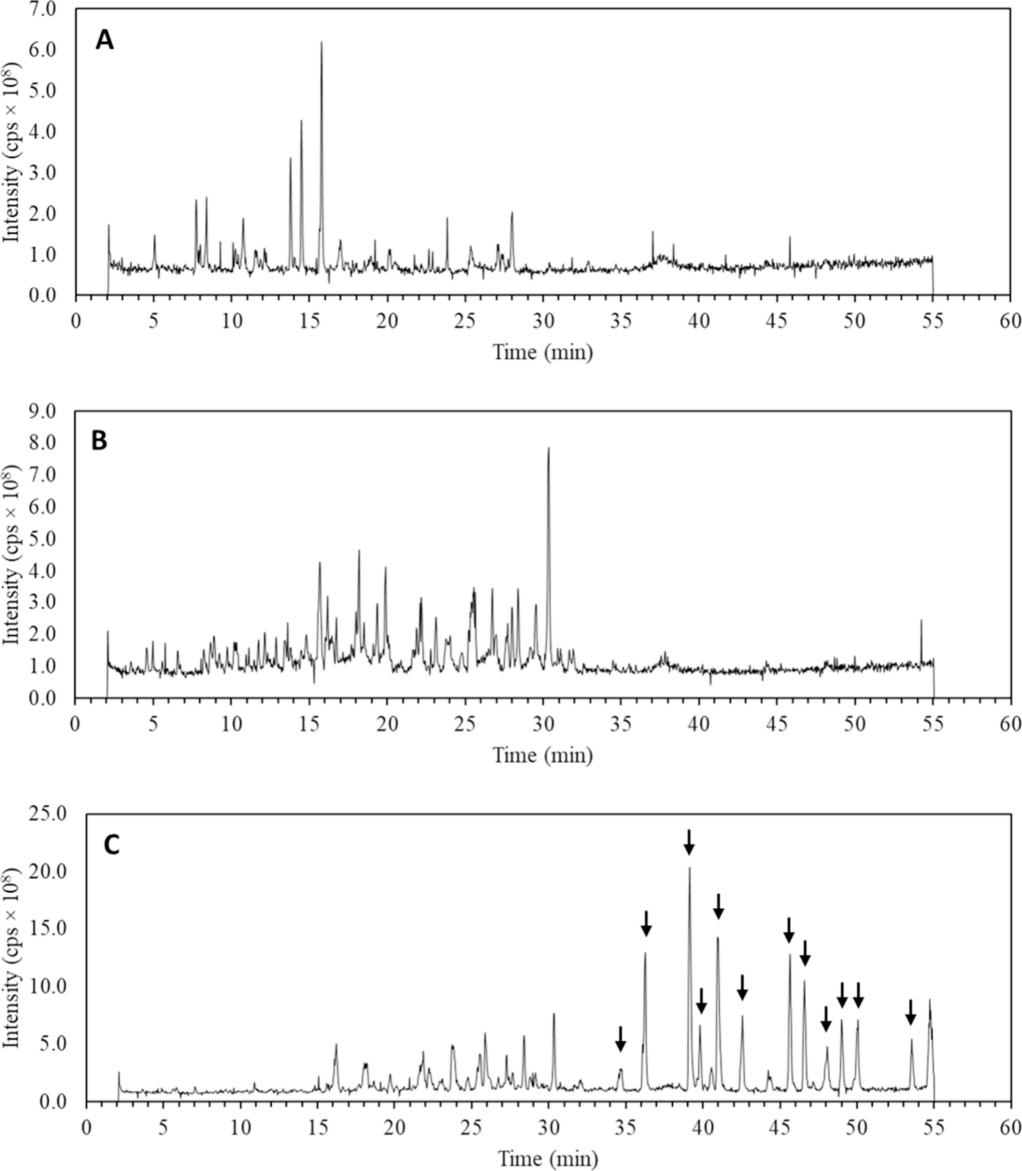
Total ion chromatograms of enhanced mass scans (EMS) of Gouda cheese (A), gastric digest (B), and intestinal digest (C) obtained by UHPLC-ESI-QTRAP-MS/MS. Arrows indicate signals corresponding to bile salts.

### 3.2 Identification of Peptides by microLC-ESI-MS/MS

In order to understand the changes in the peptide profile of Gouda cheese during simulated gastrointestinal digestion, the peptide structures were identified using microLC-ESI-MS/MS. Spectra from IDA experiments were searched against the proteome of and, in total, 54 peptide sequences released from caseins were identified in cheese and cheese digests. Ten peptides originate from α_s1_-casein, 43 from β-casein, and 1 from κ-casein (Table 1). The susceptibility of β- and α_s1_-casein to proteolysis in cheese and cheese digests during the simulated gastrointestinal digestion has been described before.^14,15^ The peptides, which were detected in the cheese sample, were indeed from β- and α_s1_-caseins (Figure 2A). This observation can be explained by the pH of Gouda cheese at around 5, which is near to the optimum for chymosin, the main proteinase from traditional rennet used in cheese manufacturing. Chymosin degrades both α_s1_- and β-caseins in the early stages of ripening.^1^ This result might also be associated with the concentration of each casein fraction in milk and consequently in cheeses. The much higher concentrations of α_s1_-casein (12–15 g/L) and β-casein (9–11 g/L) in milk, when compared to α_s2_-casein (3–4 g/L) and κ-casein (2–4 g/L), may explain the prevalence of peptides derived from α_s1_- and β-caseins.^26^ In the gastric digest, one peptide from κ-casein was also detected, besides sequences from α_s1_- and β-caseins. However, this peptide was digested during the intestinal phase of the gastrointestinal digestion; therefore, only peptides from α_s1_- and β-caseins were detected at the end of the digestion process.

**Table 1:** Amino Acid Sequences and Characteristics of Peptides Identified in Gouda Holland Cheese and during In Vitro Simulated Gastric and Intestinal Digestion^a^

mass	ion *m*/*z*	charge	peptide sequence	precursor protein	position	detected in	modification	bioactivities
862.5	432.2	2	VPSERYL	α_s1_-casein	[86–92]	cheese		ACE-inhibitory^27^
990.5	496.3	2	APFPEVFGK	α_s1_-casein	[26–34]	cheese		
1002.6	502.3	2	KKYKVPQL	α_s1_-casein	[102–109]	gastric		
1119.6	560.8	2	APFPEVFGKE	α_s1_-casein	[26–35]	cheese		
1247.7	624.8	2	APFPEVFGKEK	α_s1_-casein	[26–36]	cheese		
1266.7	634.3	2	YLGYLEQLLR	α_s1_-casein	[91–100]	intestinal		anxiolytic^28^
1384.9	693.5	2	LRLKKYKVPQL	α_s1_-casein	[99–109]	gastric		antimicrobial^29,30^
1518.8	760.4	2	PFPEVFGKEKVNE	α_s1_-casein	[27–39]	gastric		
1534.9	768.5	2	RPKHPIKHQGLPQ	α_s1_-casein	[1–13]	cheese		
1565.8	783.9	2	VPSERYLGYLEQL	α_s1_-casein	[86–98]	cheese		
801.5	802.5	1	HLPLPLL	β-casein	[134–140]	intestinal		ACE-inhibitory^31^
866.5	867.6	1	PVVVPPFL	β-casein	[81–88]	intestinal		
937.5	470.2	2	GPVRGPFPI(−0.98)	β-casein	[199–207]	intestinal	amidation	
938.5	470.3	2	GPVRGPFPI	β-casein	[199–207]	intestinal		
994.6	498.3	2	PVRGPFPII	β-casein	[200–208]	gastric		
996.6	499.3	2	VRGPFPIIV	β-casein	[201–209]	cheese		ACE-inhibitory^32^
1012.5	507.3	2	HKEMPFPK	β-casein	[106–113]	intestinal		antimicrobial,^33^ iron-chelating,^34^ antioxidant^35^
1051.6	526.9	2	GPVRGPFPII	β-casein	[199–208]	intestinal		
1093.7	547.9	2	PVRGPFPIIV	β-casein	[200–209]	gastric		
1129.7	565.9	2	LHLPLPLLQS	β-casein	[133–142]	gastric		
1130.6	566.2	2	Q(−17.03)EPVLGPVRGP	β-casein	[194–204]	cheese	pyro-glu from Q	
1142.7	572.8	2	VENLHLPLPL(−0.98)	β-casein	[130–139]	intestinal	amidation	
1203.6	602.8	2	MPFPKYPVEP	β-casein	[109–118]	cheese		ACE-inhibitory^36^
1247.7	624.9	2	PVLGPVRGPFPI	β-casein	[196–207]	intestinal		
1256.7	629.4	2	VENLHLPLPLL	β-casein	[130–140]	gastric		ACE-inhibitory^37^
1258.7	630.4	2	DVENLHLPLPL	β-casein	[129–139]	intestinal		
1263.8	632.9	2	LGPVRGPFPIIV	β-casein	[198–209]	intestinal		
1310.7	656.3	2	YQEPVLGPVRGP	β-casein	[193–204]	cheese		
1319.7	660.9	2	PVVVPPFLQPEV	β-casein	[81–92]	gastric		
1335.8	668.8	2	LPVPQKAVPYPQ	β-casein	[171–182]	cheese		
1350.7	676.4	2	MPFPKYPVEPF	β-casein	[109–119]	cheese, gastric		
1359.7	680.9	2	TDVENLHLPLPL	β-casein	[128–139]	intestinal		
1360.8	681.4	2	PVLGPVRGPFPII	β-casein	[196–208]	gastric, intestinal		
1450.8	726.4	2	PVVVPPFLQPEVM	β-casein	[81–93]	gastric		
1457.8	729.9	2	YQEPVLGPVRGPF	β-casein	[193–205]	gastric, intestinal		
1459.9	731	2	PVLGPVRGPFPIIV	β-casein	[196–209]	cheese, gastric, intestinal		
1472.8	737.5	2	TDVENLHLPLPLL	β-casein	[128–140]	gastric, intestinal		
1472.8	737.4	2	LTDVENLHLPLPL	β-casein	[127–139]	intestinal		
1479.7	740.9	2	EMPFPKYPVEPF	β-casein	[108–119]	intestinal		
1489.9	746	2	EPVLGPVRGPFPII	β-casein	[195–208]	cheese		
1504.8	753.4	2	QEPVLGPVRGPFPI	β-casein	[194–207]	intestinal		
1511.7	756.9	2	MHQPHQPLPPTVM	β-casein	[144–156]	intestinal		
1554.8	778.4	2	YQEPVLGPVRGPFP	β-casein	[193–206]	cheese		
1573.9	788	2	TLTDVENLHLPLPL	β-casein	[126–139]	intestinal		
1585.9	794	2	LTDVENLHLPLPLL	β-casein	[127–140]	gastric		
1600.9	801.5	2	Q(−17.03)EPVLGPVRGPFPII	β-casein	[194–208]	cheese	pyro-glu from Q	
1617.9	810	2	QEPVLGPVRGPFPII	β-casein	[194–208]	cheese, intestinal		
1667.9	835	2	YQEPVLGPVRGPFPI	β-casein	[193–207]	cheese, intestinal		antimicrobial^38^
1687	844.6	2	TLTDVENLHLPLPLL	β-casein	[126–140]	gastric		
1698.8	850.6	2	WMHQPHQ(+.98)PLPPTVM	β-casein	[143–156]	gastric	deamidation	
1700	851	2	Q(−17.03)EPVLGPVRGPFPIIV	β-casein	[194–209]	cheese	pyro-glu from Q	
1717	859.6	2	QEPVLGPVRGPFPIIV	β-casein	[194–209]	cheese		ACE-inhibitory^5,39^
1774	888	2	SLTLTDVENLHLPLPL	β-casein	[124–139]	intestinal		
1781	891.6	2	YQEPVLGPVRGPFPII	β-casein	[193–208]	cheese		
1880.1	941	2	YQEPVLGPVRGPFPIIV	β-casein	[193–209]	cheese, gastric, intestinal		antithrombin,^40^ antimicrobial,^38^ ACE-inhibitory^40^
1993.1	997.7	2	LYQEPVLGPVRGPFPIIV	β-casein	[192–209]	gastric		immunomodulatory^41^
1421.8	711.9	2	FSDKIAKYIPIQ	κ-casein	^18–29^	gastric		

a The table includes the peptide mass, ion *m*/*z*, charge, sequence, precursor protein, position, detection source, modifications, and bioactivities. Peptides are grouped according to their precursor proteins and are listed by mass. Bioactivities of peptides are reported according to the cited literature.

**Figure 2:**
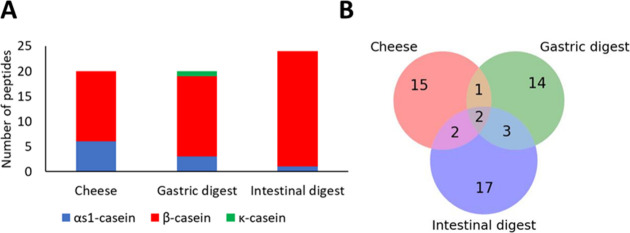
Distribution of peptides derived from α_s1_-, α_s2_-_,_ β-, and κ-casein in each sample (A) and Venn diagram (B) showing the distribution of identified peptides in cheese and digests.

The distribution of the peptides among the different samples is shown in Figure 2. Twenty peptides were identified in the cheese sample, 20 in the gastric digest, and 24 in the intestinal digest. Among the peptides detected in the present study, 14 were previously reported in a study that evaluated the digestion of Valdeón cheese, a Spanish blue-mold cheese obtained from pasteurized cow and goat’s milk.^14^ Although there was not a great difference in the number of peptides identified in cheese and digests, only 2 peptides [β-CN (f193–209) and β-CN (f196–209)] resisted digestion and were found in cheese, gastric, and intestinal digests. Similarly, a low number of cheese peptides resistant to proteolysis during simulated digestion was already reported by Sánchez-Rivera et al.,^14^ who found that 12.1% of the identified peptides were present in digested samples and Valdeón cheese before digestion.

Figures 3–5 show the location of the peptides identified in cheeses, gastric, and intestinal digests, respectively, within the amino acid sequences of the parent proteins. The cleavage sites of the main enzymes during cheese processing, ripening, and digestion are indicated in the figures. The schemes clearly show that the peptides released during cheesemaking, ripening, and digestion are not evenly distributed within the amino acid sequences of the caseins. On the contrary, there are some specific regions of each casein where more peptides were identified and were, therefore, more prone to hydrolysis by the action of enzymes present in cheese and the gastrointestinal tract.

**Figure 3:**
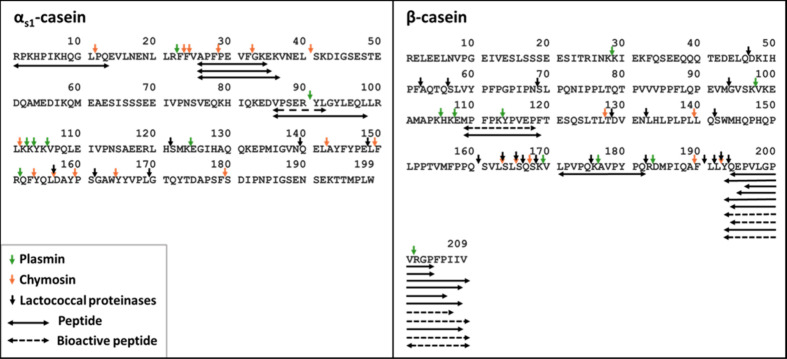
Peptide maps of Gouda cheese. Amino acid sequences of α_s1_- and β-casein are displayed in a single-letter code. Horizontal arrows represent peptides identified in the cheese sample. Dashed horizontal arrows represent the bioactive peptides identified. Vertical arrows indicate the cleavage sites of the main enzymes active in cheese ripening in each protein sequence.^4,24^

In cheese samples, α_s1_-casein-derived peptides were identified only within the sequence 1–40 and 85–98 (Figure 3). After the production of the Gouda cheese with mesophilic starter cultures and low cooking temperatures, the residual coagulant retained in the curd provides the initial proteolysis of caseins.^1^ This results in the release of large and intermediate-sized peptides, which are hydrolyzed to short peptides and amino acids by proteinases and peptidases from starter lactic acid bacteria (LAB), nonstarter LAB, and secondary cultures.^3,4^ The primary site of chymosin action on α_s1_-casein is the peptide bond F_23_–F_24_. The hydrolysis at this bond leads to the release of the peptides α_s1_-CN (f1–23) and α_s1_-CN (f24–199) that are further hydrolyzed during ripening mainly by enzymes from the starter lactic acid culture.^4^ In milk, lactocepins, which are LAB cell-envelope-associated proteinases, hydrolyze intact caseins, releasing short peptides that are taken up by the bacterial cells. The mechanisms of action of these enzymes in cheese ripening are different. The peptides released by the primary hydrolysis of caseins by the residual coagulant are the preferred substrates for the action of lactocepins.^4^ The cleavage of the peptide α_s1_-CN (f1–23) at the Q_13_–E_14_ bond by lactocepins from strains, which are commonly used in Gouda cheese manufacturing, results in the release of the peptide α_s1_-CN (f1–13) detected in the cheese sample.^1,2,4^ Plasmin, an indigenous milk proteinase, may also play an important role in cheese ripening, and its contribution to proteolysis varies depending on the cheese variety manufacturing protocol.^4^ Figure 3 shows that none of the α_s1_-casein-derived peptides can be explained by plasmin activity (plasmin cleavage sites indicated by green vertical arrows).

Figure 3 also shows β-casein-derived peptides identified in cheese samples. A clear prevalence of peptides from the C-terminal region of β-casein was observed. During the early stages of ripening, chymosin cleaves β-casein at the bound L_192_–Y_193_, leading to the release of β-CN­(f193–209), which is commonly detected in cheese since the beginning of ripening.^4,42,43^ This oligopeptide is further hydrolyzed by the action of lactococcal endopeptidases in the cleavage sites P_204_–F_205_, P_206_–I_207_, I_207_–I_208_, associated with the release of several peptides from the C-terminal sequence of β-casein identified in the present study.^44^

Peptides identified in the gastric digest are shown in Figure 4. Only 3 peptides from α_s1_-casein were identified in the gastric digest [α_s1_-CN (f102–109), α_s1_-CN (f99–109), and α_s1_-CN (f27–39)]. These peptides were not detected in the cheese samples, and the release of most of these peptides can be explained by pepsin cleavage sites (blue vertical arrows). These peptides were previously detected in the gastric digest obtained after the simulated gastrointestinal digestion of yogurt.^39^

**Figure 4:**
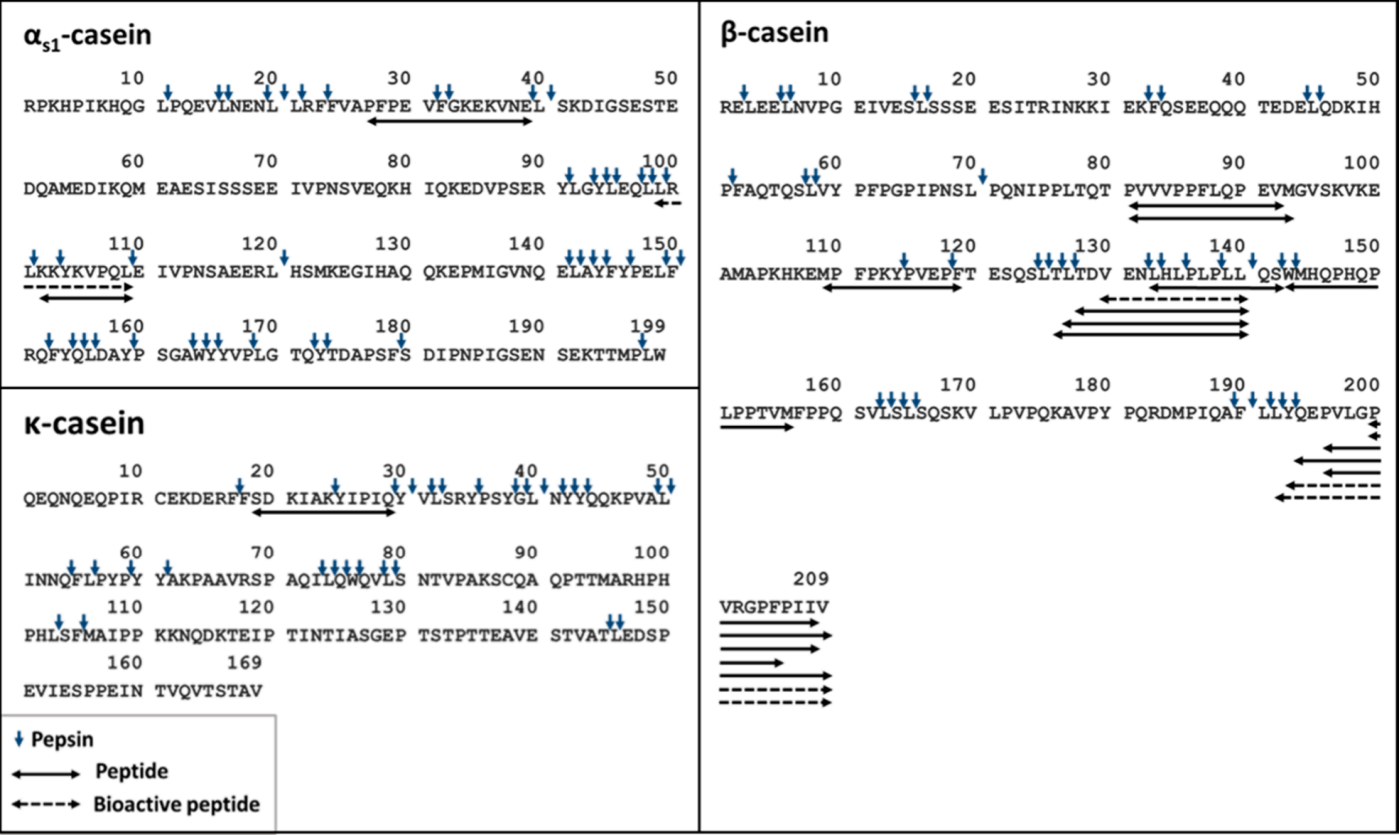
Peptide map of the simulated gastric digest of Gouda cheese. Amino acid sequences of α_s1_-, β-, and κ-casein are displayed in a single-letter code. Horizontal arrows represent peptides identified in the gastric digest. Dashed horizontal arrows represent the bioactive peptides identified. Vertical arrows indicate the cleavage sites of pepsin in each protein sequence.^25^

One peptide from κ-casein [κ-CN (f18–29)] was identified in the gastric digest (Table 1). It is worth noting that during the cheese production, κ-casein is hydrolyzed by the action of milk-clotting enzymes at the bond F_105_–M_106_, which leads to the casein micelle’s destabilization and the subsequent milk coagulation. The glycomacropeptide, representing the residue from amino acids 106–169, is released and removed with the whey during the draining of the curd, which explains the absence of peptides derived from this part of the protein.^45^

Sixteen peptides of β-casein were identified in the gastric digest. Some peptides between amino acids at positions 126 and 156 that were not detected in cheese samples were detected in the gastric phase. The release of these peptides is indeed associated with pepsin activity, as demonstrated by the presence of several cleavage sites of this digestive enzyme in this sequence (Figure 4). As already observed in the cheese sample, a clear prevalence of peptides from the C-terminal fraction of β-casein was detected. This predominance of peptides from this particular area is probably due to the combination of two factors: (i) the absence of pepsin cleavage sites between the amino acids 194 and 209, which contributes to the stability of peptides from this C-terminal fraction released during cheese processing and ripening, and (ii) the action of pepsin during gastric digestion due to the presence of this enzyme cleavage sites on L_191_–L_192_ and L_192_–Y_193_. The action of pepsin on these cleavage sites may be associated with the further release of β-CN (f193–209), already present in cheese, and the release of β-CN (f192–209).

Peptides identified in the intestinal digest are listed in Figure 5. Only 1 peptide from α_s1_-casein was identified in the intestinal digest [α_s1_-CN (f91–100)], which was not detected in cheese and gastric digest. The release of this peptide during simulated intestinal digestion of yogurt was previously reported^39^ and can be explained by the activity of trypsin at cleavage sites R_90_–Y_91_ and R_100_–L_101_ (Figure 5).

**Figure 5:**
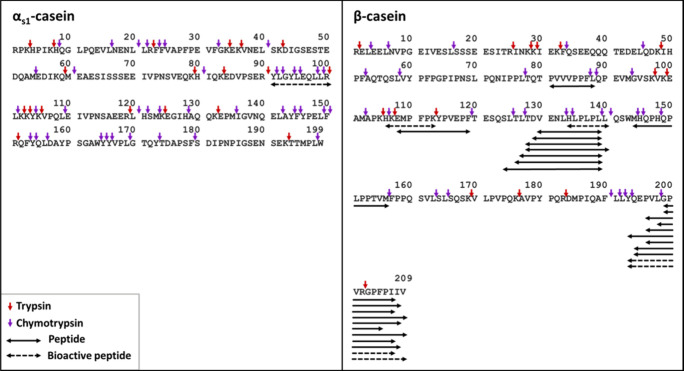
Peptide map of the simulated intestinal digest of Gouda cheese. Amino acid sequences of α_s1_- and β-casein are displayed in a single-letter code. Horizontal arrows represent peptides identified in the intestinal digest. Dashed horizontal arrows represent the bioactive peptides identified. Vertical arrows indicate the cleavage sites of trypsin and chymotrypsin in each protein sequence.^25^

β-Casein-derived peptides were the great majority of peptides detected after the intestinal phase of simulated digestion. As already observed for cheese and gastric digests, the β-casein peptides identified in the intestinal digest were not distributed homogeneously throughout the protein amino acid sequence. Most of the β-casein peptides detected in this phase of digestion were located between amino acids 124 and 140, or in the C-terminal fraction between amino acids 192 and 209.

β-casein fragments PVLGPVRGPFPIIV [β-CN (f196–209)] and YQEPVLGPVRGPFPIIV [β-CN (f193–209)] were detected in cheese and at all stages of digestion, suggesting resistance to gastrointestinal digestion. However, the C-terminal region of β-casein was found to be more susceptible to proteolysis, leading to the degradation of these peptides, while they are also continuously released from the protein by digestive enzymes throughout all stages. In the case of β-CN (f193–209), this hypothesis is supported by the presence of pepsin and chymotrypsin cleavage sites at the L_192_–Y_193_ bond (Figures 4 and 5). Moreover, findings from our research suggest that the C-terminal region of the β-casein is highly susceptible to proteolysis, as peptides from this region have been identified in raw milk, as well as after thermal processing, storage, and fermentation.^21,46–49^ Notably, β-CN (f196–209) and β-CN (f193–209) have been consistently observed under different conditions, indicating that their persistence is likely due to simultaneous degradation and regeneration rather than inherent resistance to digestion. Furthermore, three peptide sequences were released from precursors during the gastric phase: PVLGPVRGPFPII [β-CN (f196–208)], YQEPVLGPVRGPF [β-CN (f193–205)], and TDVENLHLPLPLL [β-CN (f128–140)]—were detected in both gastric and intestinal digestions but not in cheese, suggesting that they are formed during digestion and remain present throughout the process (Table 1).

### 3.3 Bioactive Peptides in Cheese and Cheese Digests

The peptide sequences identified in this study were searched for known bioactive effects using available databases. Accordingly, 12 out of 54 peptides were associated with a bioactivity (Table 1 and Figures 3–5). Most of the bioactive peptides identified in this study had angiotensin-converting enzyme (ACE)-inhibitory activity. Six ACE-inhibitory peptides were identified in the samples [(α_s1_-CN (f86–92), β-CN (f134–140), β-CN (f201–209), β-CN (f109–118), β-CN (f130–140), and β-CN (f194–209)]. Additionally, one anxiolytic peptide [α_s1_-CN (f91–100)], two antimicrobial peptides [α_s1_-CN (f99–109), β-CN (f193–207)], and one immunomodulatory peptide [β-CN (f192–209)] were determined. Finally, two multifunctional peptides were detected: the antimicrobial, iron-chelating, and antioxidant peptide [β-CN (f106–113], and the antithrombin, antimicrobial, and ACE-inhibitor peptide β-CN (f193–209). It is worth noting that the prevalence of ACE-inhibitor peptides in the samples could be biased because this specific biological activity is the most studied bioactivity associated with milk peptides.^50^ Further studies using bioactivity-guided fractionation or virtual screening, complementary to database search, are required to investigate if additional and so far unknown bioactivities are associated with the identified peptides. Most of the bioactive peptides identified in the evaluated samples are derived from the C-terminal fraction of β-casein: β-CN (f192–209), β-CN (f193–209), β-CN (f194–209), and β-CN (f201–209). The predominance of bioactive peptides in this region is associated with the structure of their amino acid sequence, which determines their bioactivity. For instance, the antihypertensive potential is usually assessed by the ACE-inhibitory activity in vitro.^50^ ACE, a major regulator of blood pressure, is part of the renin-angiotensin system, which regulates peripheral blood pressure by the catalysis of two reactions: the conversion of angiotensin I to angiotensin II, a potent vasoconstrictor; and the degradation of bradykinin, a vasodilator peptide. Both reactions lead to blood vessel contraction and an increase in blood pressure.^51,52^ Thus, peptides that inhibit ACE activity have a potential antihypertensive effect. ACE binding and its consequent inhibition by a substrate or competitive inhibitor depend on the amino acid sequence at the three C-terminal positions. ACE preference for substrates with hydrophobic amino acids at the C-terminal tripeptide sequence might explain the ACE-inhibitory activity of peptides derived from this fraction of β-casein.^53^

Among the bioactive peptides identified in the present study, only one peptide [β-CN (f193–209)] was detected in the cheese and all phases of the simulated digestion. Four bioactive peptides identified in the cheese were not detected in the digests, which may be associated with the hydrolysis of these biologically active fragments during in vitro digestion. Six bioactive sequences were not identified in the cheese samples, but were identified in at least one digest. The appearance of these bioactive peptides in the gastric and intestinal digests indicates that gastrointestinal digestion also plays an essential role in the release of bioactive peptides. These findings reinforce the importance of including in vitro digestion models in studies aimed at evaluating the bioactive potential of cheeses, as they enable the identification of peptides present in cheese samples that may not resist digestion and the identification of peptides that might not be initially present in the food matrix but are released and potentially functional after digestion.

The identification of the bioactive peptide β-CN (f193–209) in cheese and all digests demonstrates its potential physiological effect after the consumption of cheese. The persistence of this particular bioactive peptide to the gastrointestinal digestion of fermented dairy products was demonstrated by in vitro studies that simulated the gastrointestinal digestion of yogurt and kefir samples.^39,54^ Additionally, this peptide had already been detected in the blood plasma of individuals who consumed Parmigiano Reggiano cheese, suggesting not only its resistance to the gastrointestinal tract but also its effective absorption in the human body.^55^

Additionally, six other bioactive peptides identified in the present study were previously reported in hydrolysates obtained after in vitro simulated digestion of fermented dairy products.^14,39^ The peptides α_s1_-CN (f91–100), β-CN (f134–140), β-CN (f194–209) and β-CN (f192–209) were previously identified in the intestinal digest of yogurt; the peptide β-CN (f130–140) was previously reported in the intestinal digest of Valdeón cheese; and the peptide β-CN (f106–113) in the intestinal digest of Valdeón cheese and yogurt.^14,39^ Thus, it can be assumed that these peptides are of general relevance to the bioactivity of fermented dairy products.

The present study investigated the peptide profile of young Gouda Holland cheese during the simulation of gastrointestinal digestion. We could demonstrate that the peptide profile changes with digestive enzymes action. Enzymatic hydrolysis leads to the release of 6 bioactive peptides in the gastrointestinal tract that were not present in the cheese. The identification of bioactive peptides at the end of the simulated gastrointestinal digestion reveals potential physiological effects after the consumption of Gouda cheese. Based on our results, future studies could now be conducted to investigate the resorption of these peptides in vivo and the physiological effects of Gouda cheese-derived peptides.

## Supplementary Material



## Notes

Total ion chromatograms of enhanced mass scans (EMS) of three different batches of Gouda cheese (A, B, and C) from the same brand evaluated in the study (PDF)

## Abbreviations

ACE: angiotensin-converting enzyme
ACN: acetonitrile
CN: casein
EMS: enhanced mass spectrum
EPI: enhanced product ion
ER: enhanced resolution
ESI: electrospray ionization
FA: formic acid
FDR: false discovery rate
IDA: information dependent acquisition
LAB: lactic acid bacteria
LC: liquid chromatography
LIT: linear ion trap
MBPDP: Milk bioactive peptide database
microLC-ESI-MS/MS: microliquid chromatography hyphenated electrospray ionization tandem mass spectrometry
UHPLC-ESI-MS: ultrahigh performance liquid chromatography hyphenated electrospray ionization mass spectrometry.
