# Supplementary material for: Peptide Profiling of Young Gouda Holland Cheese and Its Digests Obtained by In-Vitro-Simulated Gastrointestinal Digestion
Source: J Agric Food Chem. 2025 Aug 11;73(33):21048–58. doi: 10.1021/acs.jafc.5c04616 (PMC12371890; doi:10.1021/acs.jafc.5c04616)
Supplement: Supplementary file 1 [file jf5c04616_si_001.pdf]

Supporting information

Peptide profiling of young Gouda Holland cheese and its digests obtained by in vitro simulated gastrointestinal digestion

Débora Parra Baptista[a,b*], Sevim Dalabasmaz[a], Sabrina Gensberger-Reigl[a], Mirna Lúcia Gigante[b], Monika Pischetsrieder[a]

[a]Food Chemistry, Department of Chemistry and Pharmacy, Friedrich-Alexander-Universität Erlangen-Nürnberg (FAU), Nikolaus-Fiebiger-Straße 10, 91058, Erlangen, Germany.

[b]Department of Food Engineering and Technology, School of Food Engineering, Universidade Estadual de Campinas, UNICAMP, 13083-862, Campinas, SP, Brazil.

*Corresponding author at: Department of Food Engineering and Technology, School of Food Engineering, Universidade Estadual de Campinas, UNICAMP, 13083-862, Campinas, SP, Brazil;

E-mail: deborapb@unicamp.br

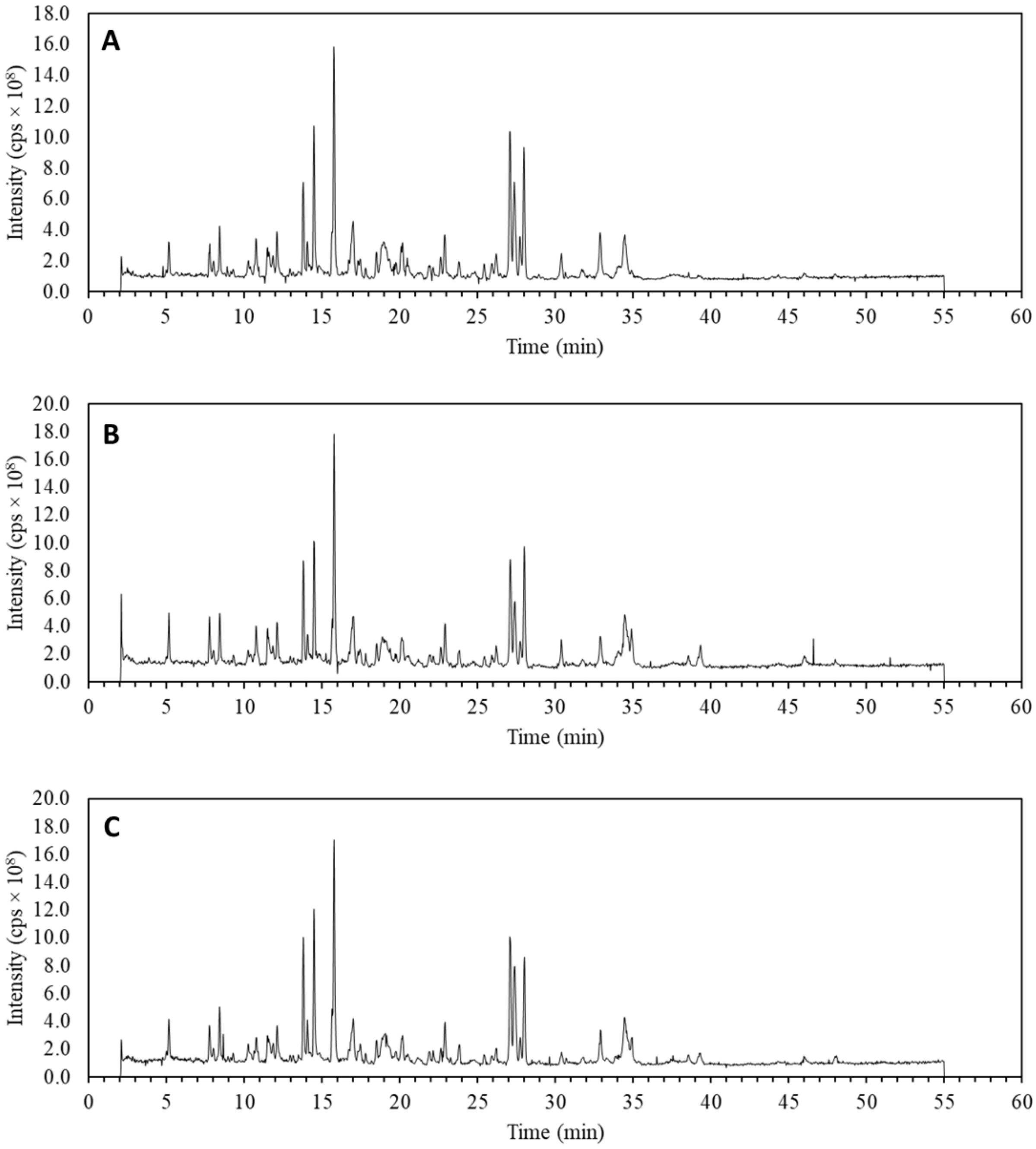

Figure S1. Total ion chromatograms of enhanced mass scans (EMS) of three different batches of Gouda cheese (A, B, and C) from the same brand evaluated in the study. The samples were prepared as described in the materials and methods section but with a different dilution factor, 2 instead of 8.
